# Graphene and Carbon Quantum Dot-Based Materials in Photovoltaic Devices: From Synthesis to Applications

**DOI:** 10.3390/nano6090157

**Published:** 2016-08-25

**Authors:** Sofia Paulo, Emilio Palomares, Eugenia Martinez-Ferrero

**Affiliations:** 1 Institut Català d’Investigació Química (ICIQ), The Barcelona Institute of Science and Technology, Avda. Països Catalans 16, Tarragona 43007, Spain; spaulo@iciq.es; 2 Fundació Eurecat, Avda. Ernest Lluch 36, Mataró 08302, Spain; 3 ICREA, Passeig Lluís Companys 23, Barcelona 08010, Spain

**Keywords:** quantum dots, graphene, carbon, photovoltaics, solar cells

## Abstract

Graphene and carbon quantum dots have extraordinary optical and electrical features because of their quantum confinement properties. This makes them attractive materials for applications in photovoltaic devices (PV). Their versatility has led to their being used as light harvesting materials or selective contacts, either for holes or electrons, in silicon quantum dot, polymer or dye-sensitized solar cells. In this review, we summarize the most common uses of both types of semiconducting materials and highlight the significant advances made in recent years due to the influence that synthetic materials have on final performance.

## 1. Introduction

Converting solar energy efficiently into either electrical or fuel sources remains one of mankind’s biggest challenges [1]. Despite the rapid progress that has been made in recent years in research into third generation solar cells, silicon is still the biggest and most important player in the PV industry. Even so, such new technologies as mixed halide perovskite solar cells are quickly catching-up in efficiency (the current record of efficiency is above 22% at 1 sun) [2,3]. Dye-sensitized and organic solar cells (which include polymer- and small organic molecule-based solar cells) have already shown their potential for applications like building integrated photovoltaics. Whereas semiconductor-based quantum dots, typically composed of cadmium or lead derivatives, have such excellent optical properties that they have been used in a wide array of optoelectronic devices such as solar cells, light emitting diodes, bioimaging or optical sensors [4,5,6].

In this context, carbon-based quantum dots have emerged as potential candidates for application in such devices. Since their discovery early 2000s, carbon-based quantum dots have been the focus of intensive research because of their excellent luminescent properties, good solubility and biocompatibility [7,8]. This research effort increased exponentially after the Nobel Prize awarded to Novoselov and Geim for discovering graphene and describing its properties [9].

**Figure 1:**
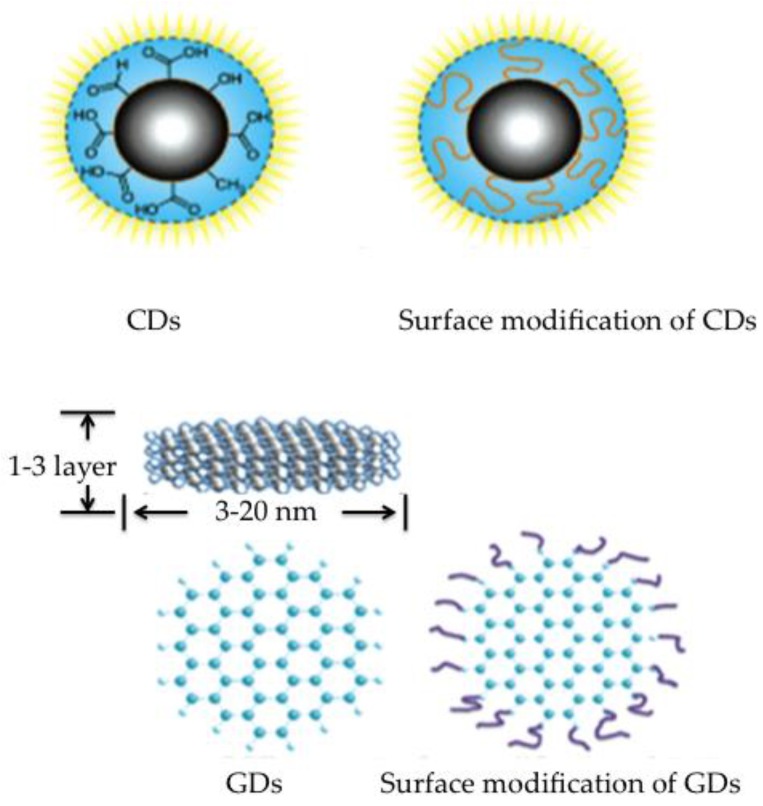
Illustration of CD (top) and GD (bottom) structures. Reproduced with permission of [12,13,14].

These carbon-based nanostructures are in fact two different allotropes (Figure 1). On the one hand, carbon quantum dots (CDs) are quasi-spherical nanoparticles less than 10 nm in diameter, formed by crystalline sp2 graphite cores, or amorphous aggregations, which have a quantum confinement effect. On the other hand, there are dots—the so-called graphene quantum dots (GDs)—made up of single or very few graphene lattices (<10) that have quantum confinement effect and edge effects. GDs are usually more crystalline than CDs because their conjugated domains are larger and their structure regular. Both allotropes are functionalized with complex surface groups, specially oxygen-related molecules such as carboxylates or hydroxylate derivatives that remain after the synthetic procedure and enhance the optical properties and the solubility of the particles [10,11]. It should be pointed out that variability in the fabrication of these materials results in different surface functionalization and the addition of complexity to the hybridization of the carbon atoms.

Carbon-based dots have many advantages over non-carbon dots because of their chemical inertness and lower citotoxicity photobleaching and cost. For instance, they can be produced from biomass. In recent years, carbon-based dots have been tested as fluorescent probes, in light emitting diodes, solar cells, biosensors, supercapacitors, lithium ion batteries and catalysts [15,16,17,18,19,20] and have even been combined with non-carbon dots in optoelectronic applications [21]. Despite their excellent optical properties, they have not performed in photovoltaics as well as non-carbon based quantum dot solar cells. As far as we know, no exhaustive review has been made of carbon-based dots used in photovoltaics. Therefore, in an attempt to understand why these nanostructures have so far failed to realize their potential, in this review we analyze the main achievements in the link between functionality and the synthesis of the material. We aim to give a general overview of how these promising carbon nanostructures can be applied in PV dividing this feature article into the following parts:

1. General synthetic approaches.
2. Photonic properties.
3. Graphene quantum dots in photovoltaic devices.
4. Carbon quantum dots in photovoltaic devices.
5. Outlook and perspectives.

At the end of the manuscript, we have included a list of the abbreviations used throughout the text and Table 1 and Table 2 summarize the research done on graphene and carbon quantum dots in photovoltaics, respectively.

## 2. General Synthetic Approaches

**Figure 2:**
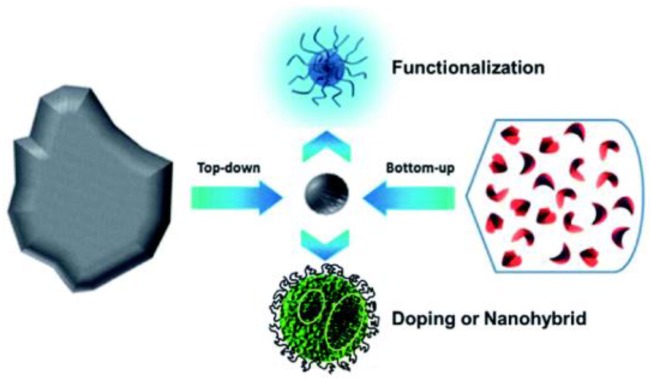
Schematic representation of both synthetic approaches. Reproduced with permission of [16].

Numerous papers describe synthetic procedures for preparing carbon and graphene quantum dots. Two main approaches can be distinguished: bottom-up and top-down synthesis. The bottom-up route builds nanostructures from small organic molecular precursors by pyrolysis, combustion or hydrothermal methods while the top-down approach is based on cutting small sheets via physical, chemical or electrochemical techniques until the required particle size is reached (Figure 2). In both cases, post treatment is done to purify or modify the surface functionalization and improve the performance of the dots. For example, the quantum yield increases after surface passivation of CDs or functionalization because the emissive traps on the nanoparticle surface disappear. Likewise, doping with heteroatoms such as nitrogen and phosphor, or metals such as Au or Mg improves the electrical conductivity and solubility of CDs and GDs [24,27].

In this review, we have focused exclusively on the synthetic procedures described for carbon-based dots applied in photovoltaic devices. Of course, other excellent reviews on the vast number of applications of carbon and graphene quantum dots can be found in [16,17,51], and the references cited therein. 

### 2.1. Bottom-up Approach

#### 2.1.1. Hydrothermal/Solvothermal Synthesis

Hydrothermal synthesis is a widespread procedure that consists of a one-step synthetic technique in which an organic precursor is heated in a Teflon line to achieve high temperature and pressure. Using various organic precursors and modifying the temperature, the optoelectronic properties of the dots are tuned. It is, thus, a low-cost, non-toxic method. In addition, hydrothermal methods produce dots with a diameter of 10 nm, which are bigger than dots produced by other techniques such as electrochemical preparations (3–5 nm).

**Figure 3:**
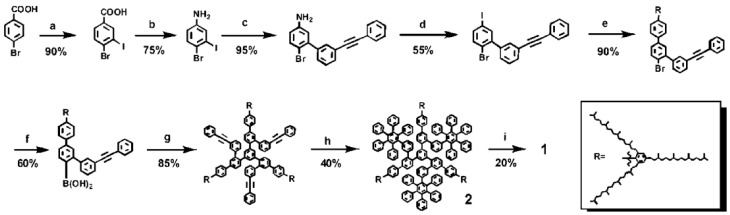
Suzuki reaction followed to prepare graphene dots (described as product number 1 in the reaction scheme) from bromobenzoic acid. Reproduced with permission of [36]. Steps are as follows: (**a**) NaIO_4_, I_2_, concentrated H_2_SO_4_, room temperature; (**b**) Heated with diphenylphosphoryl azide in triethylamine and tert-butanol at 80 °C, followed by treatment with CF_3_COOH in dichloromethane at room temperature; (**c**) Suzuki condition with 3-(phenylethynyl)phenylboronic acid, Pd(PPh_3_)_4_, K_2_CO_3_ in water, ethanol, and toluene mixture, 60 °C; (**d**) Iodine and tert-butyl nitrite in benzene, 5 °C to room temperature; (**e**) Suzuki condition with substituted phenyl boronic acid, Pd(PPh_3_)_4_, K_2_CO_3_ in water, ethanol, and toluene mixture, 80 °C; (**f**) Treatment with butyllithium in tetrahydrofuran (THF) at −78 °C, then with triisopropyl borate at −78 °C, followed by treatment with acidic water at room temperature; (**g**) Suzuki condition with 1,3,5-triiodobenzene, Pd(PPh_3_)_4_, K_2_CO_3_ in water and toluene mixture, 80 °C; (**h**) Tetraphenylcyclopentadienone in diphenylether, 260 °C; (**i**) FeCl3 in nitromethane and dichloromethane mixture, room temperature.

Pioneering work by Mirtchev and coworkers introduced the use of carbon quantum dots as sensitizers in dye-sensitized solar cells (DSSC) prepared by dehydrating ɣ-butyrolactone [22]. In contrast, Yan et al. synthesized graphene dots from bromobenzoic acid using well-known Suzuki-Miayura reaction conditions (Figure 3). In order to prepare large graphene dots and avoid aggregation, they covalently attached 1,3,5-trialkyl phenyl moieties to the edge of the graphene, shielding them in the three dimensions [36]. Last but not least, Zhang et al. prepared the CDs from citric acid and ethylenediamine in aqueous solution heated for 10 h at 250 °C obtaining uniform 1–2 nm size particles [23], whereas Liu et al. synthesized the CDs combining polystyrene-co-maleic and ethylenediamine dissolved in DMF at 200 °C for 5 h [26].

The nanoparticles are nitrogen doped by this route as well. Zhang et al. used carbon tetrachloride and sodium amide as starting materials and methylbenzene as the solvent, heating at 200 °C for different periods of time to prepare well-dispersed crystalline CDs. By controlling the reaction time, the authors tuned the size and the nitrogen content of the dots in such a way that prolonged reaction times favored the incorporation of nitrogen into the carbon framework and the increase in the particle size. Regardless of the reaction time, the dots had amino functional groups on their surface [24].

#### 2.1.2. Microwave Irradiation Synthesis

As well as the speed of the synthesis, another important advantage that microwave synthetic methods have over hydrothermal synthesis is that they can be used at lower temperatures. Dao et al. obtained high quality CDs by this synthetic approach. They mixed citric acid and urea in distilled water and the solution was then heated in a microwave oven at 700 W for 4 min. The supernatant was neutralized with sodium bicarbonate and cleaned with distilled water [27]. The resulting dots were doped with Au by chemical reduction of HAuCl_4_ with formic acid to prepare three dimensional raspberry-like particles with a diameter of 200 nm formed by gold branches that originated high surface areas. In addition, Tsai et al. synthesised water soluble GDs by microwave irradiation using glucose as the carbon source and water as solvent heating at 700 W for 11 min. The as-prepared dots measured 3.4 nm in diameter, as observed by AFM (Atomic Force Microscopy) and TEM (Transmission Electron Microscopy) [37].

#### 2.1.3. Soft Template Method

In this approach, reported by Kwon et al., CDs are made into an emulsion that acts as a self-assembled soft template because the size of the dots is controlled by regulating the amount of the emulsifier. Synthesis in a non aqueous medium favors organic-based surface capping and size tuning. For that, the authors mix oleylamine and octadecene with citric acid solved in water. The water droplets, stabilized by the oleylamine, are eliminated heating at 250 °C forcing the intermolecular dehydration of citric acid molecules which form polymer-like structures. Further carbonization render organic soluble carbon dots capped by oleylamine molecules that are chemically bound to the dot surface carbonyl groups. The concentration of oleylamine determines the final size of the dots [32].

### 2.2. Top-down Approach

#### Electrochemical Methods

**Figure 4:**
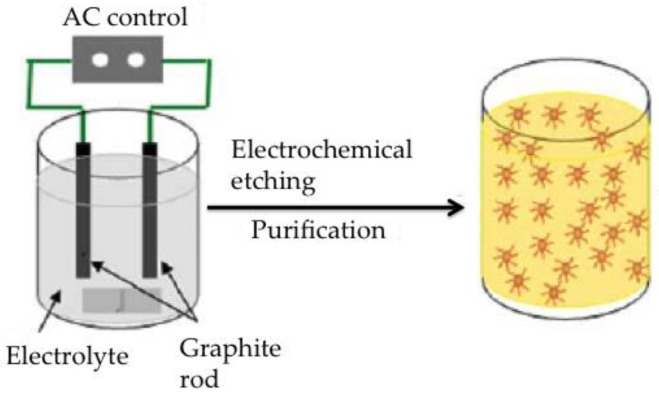
Schematic view of the obtention of CDs by electrochemical methods. Reproduced with permission of [28].

Electrochemical methods make it possible to fine tune carbon nanostructures by controlling the voltage/current applied. For instance, applying a controlled bias to a bulk of carbon precursors leads to electrochemical corrosion reactions over the carbon reactants and subsequently to carbon nanostructures. It is important to notice that this particular technique does not require high temperatures, is easy to scale-up and can proceed under aqueous or non-aqueous solutions. It is one of the fastest routes for preparing graphene sheets [52]. For example, Sun et al. prepared carbon quantum dots by combining the electrochemical method with etching methods [28]. In brief, they used graphite rods as both electrodes whereas the reaction was conducted by applying an alternate bias between 100 and 150 V during 10 h in the presence of an ethanol solution of NaOH (Figure 4). Then they added MgSO_4_ followed by stirring, deposition, centrifugation and drying of the solvent in order to obtain the uniform and monodisperse dots.

More recently, Zhu and co-workers obtained graphene dots. In this case, the electrolysis took place under a current intensity between 80 and 200 mA/cm^2^ with a graphite rod as anode in a basic solution and Pt foil as the counter electrode. In order to finish the reaction, they added 1 mL of 80% hydrazine hydrate and stirred the solution for 8h. It was then centrifuged and dialyzed in water for one day [38,53]. Yan Li and co-workers prepared homogeneous GDs by electrochemical methods from graphene films [39]. For that, graphene films, prepared by the filtration method and treated in oxygen plasma to improve its hidrophilicity, were used as working electrodes in combination with Pt wire and Ag/AgCl that acted as counter and reference electrode, respectively, in phosphate buffer solution. After CV scan rate of 0.5 V/s within ±3 V in 0.1 M PBS, water soluble GDs with uniform 3–5 nm size were obtained.

### 2.3. Acidic Oxidation or Chemical Ablation

In essence, this two-step procedure consists of the exfoliation of graphite powder using concentrated mineral acids and oxidizing agents under refluxing conditions. This approach, also known as Hummers method, is one of the most popular procedures described for obtaining graphite oxide. The first step is often followed by further chemical reduction to prepare the quantum dots. For example, Dutta et al. treated graphite with sodium nitrate in aqueous sulfuric acid solution with potassium permanganate stirring for four days. Once the graphite oxide was ready, ultrasonication of the sample in water produced graphene oxide, which was converted to graphene dots by reduction in hydrazine solution [40]. Pan et al. prepared GDs from graphene oxide that was transformed to graphene sheets by the Hummer’s method. For the second step, they applied a hydrothermal treatment in basic solution (heating at 200 °C for 10 h at pH 8) to cut the graphene sheets into dots that were further purified by dyalisis [43] .

Carbon black has also been used as a carbon source. Chen et al. prepared GDs by oxidation of the carbon black in nitric acid under reflux conditions overnight. After cooling and centrifugation, the supernatant was heated to recover the dots [44]. An alternative source are carbon fibers, as reported by Peng et al. [49]. In this case, the fibers were sonicated and heated for 30 h at 100 °C in acidic medium. After being cooled, the mixture was diluted in water, the pH tuned to 7 and the solution dialyzed.

## 3. Photonic Properties

**Figure 5:**
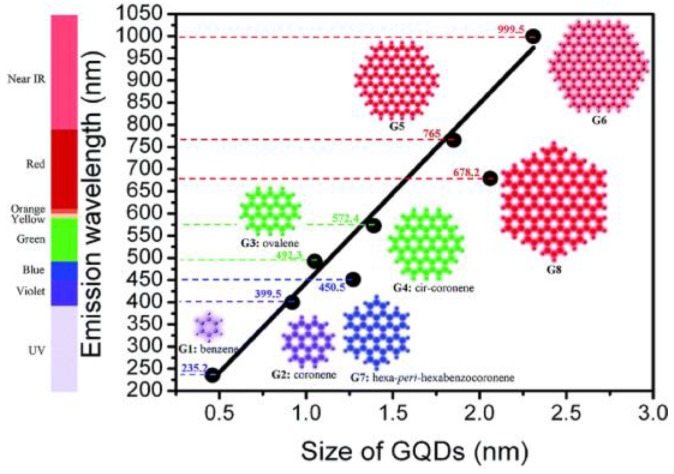
Estimated variation of the emission wavelength with the size for GDs. Reproduced with permission of [54].

It is a remarkable fact that both structures show quantum confinement effects, which means that the energy band gap is determined by the size and shape of the structure (Figure 5). In addition, the optical properties are also influenced by the fabrication variability, which results in a wide array of sizes and surface functionalizing groups and/or defects. Therefore, the determination of the origin of the material’s optical properties is one of the most controversial topics in research into carbon and graphene quantum dots. 

### 3.1. Light Absorption

Both CDs and GDs have an absorbance band in the UV region between 260 and 320 nm assigned to the π–π* transition of C=C bonds with sp2 hybridization and, sometimes, a weaker shoulder at 270–400 nm attributed to δ–π* transitions of the C=O bonds, with a tail extending into the visible wavelengths. Graphene quantum dots also have extinction coefficients in the UV region from 10 to 200 × 10^3^ M^−1^cm^−1^, which is larger than common fluorophores and comparable to other quantum dots [53,54].

### 3.2. Light Emission

The photoluminescence (PL) mechanism in CDs and GDs is still an open question and different processing methods cause PL of different origins. In fact, PL has been reported to be influenced by the dot size, the excitation wavelength, the degree of surface functionalization or oxidation, the pH during synthesis, the solvent polarity and the doping with heteroatoms. Both CDs and GDs show strong photoluminescent emission that is mostly exciton depedent, which means that the emission peak moves as the excitation wavelength is changed. The origin of fluorescence emission has been intensively studied and assigned to quantum confinement effects, triplet carbenes at zigzag edges or edge defects, excitonic transitions, surface states or functional groups [55,56,57,58,59].

## 4. Graphene Quantum Dots in Photovoltaics

Researchers have already found various applications for graphene dots in solar cells, mainly in silicon-based solar cells, dye-sensitised solar cells, organic solar cells (OSC) and, more recently, perovskite solar cells. Silicon diodes (either crystalline, c-Si, or amorphous, a-Si) are based on silicon p-n junctions that act both as light absorbers and charge transport carriers. Although Si diodes dominate the PV market because of their high efficiency (recently reported to be 25.6%) [2] and long lifetime, the incorporation of graphene sheets as transparent electrodes has already been explored to improve the performance of the diodes [60]. 

**Figure 6:**
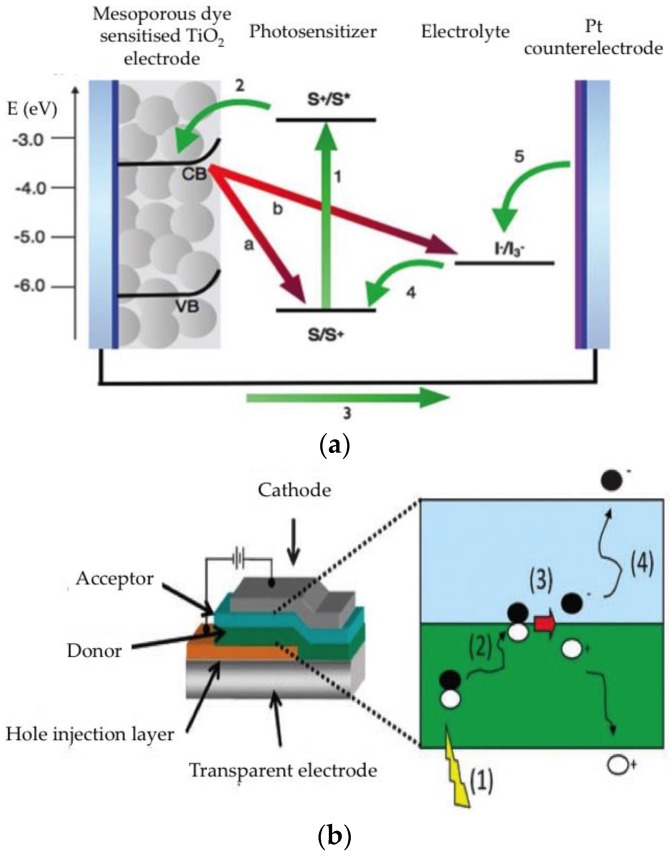
Schematic representation of the composition and charge transfer processes in (**a**) DSSC [(1) light absorption; (2) electron injection; (3) electron collection; (4) reduction of the oxidized dye cation by the redox couple; (5) regeneration of the electrolyte at the counterelectrode] and (**b**) OSC [(1) Light absorption and creation of an exciton; (2) exciton diffusion; (3) exciton splitting at the interface; (4) diffusion and collection of charges]. Reproduced with permission of [61,65], respectively.

The device structure of DSSC, which are photo-electrochemical solar cells, is more complex. The electron transport layer is often based on mesoporous nanocrystalline metal oxide films, usually TiO_2_ or ZnO, supported on a conducting substrate. The electron transport layer can be configured as planar, mesoporous or columnar morphologies. The mesoporous metal oxide film is sensitized to absorb visible light after the adsorption of a dye monolayer. Examples of popular dyes are Ru(II)-containing polypyridyls, porphyrins, phthalocyanines, squarines or organic dyes [61]. The device is filled with an electrolyte that regenerates the sensitizer, normally iodide/tri-iodide redox electrolyte, defined as hole transport layer (HTL) and a platinum coated counter electrode (Figure 6a). DSSCs have attracted considerable attention since the landmark paper in 1991 by Gratzel and O’Regan [62]. Because of their potential low cost, environmentally friendly components, ease of fabrication in air and such optical properties as transparency and colour, which depends on the dye selected, DSSCs have attracted attention for building-integrated photovoltaic applications. Record efficiencies of 13% have recently been achieved with the molecularly engineered porphyrin dye SM315 [63]. A solid state version of the DSSC can be achieved by replacing the liquid electrolyte with a solid hole transport material such as spiro-OMeTAD or a semiconductor polymer [64].

Organic photovoltaics (OSCs) combine carbon-based semiconductor materials and molecules, which play the roles of light absorption and carrier transport sandwiched between selective metal electrodes. Depending on the molecular weight of the organic material, OSCs are classified as polymer (PSC) or small-molecule solar cells (SMOPV). The former are processed from solution in organic solvents to form bulk heterojunctions in the photoactive layer in conjunction with either the electron or hole acceptor material. The latter can also be processed using high vacuum techniques. It is well established that the intermixing of the donor and the acceptor optimizes the exciton separation and subsequent carrier collection (Figure 6b) [65,66]. For many years, the most efficient, and widely used, electron acceptor materials were those based on such fullerene derivatives as PCBM. In fact, record efficiencies above 11% have recently been reported using PffBT4T-derivatives as donors and C71-fullerene derivatives as acceptors [67,68]. Only recently has it been shown that other electron acceptor materials can be used to match the high efficiency obtained with fullerene derivatives [69].

In both types of solar cell, DSSC and OSC, which differ from silicon solar cells in the materials they use to transport the hole and the electron carriers, carbon nanomaterials can be easily adapted to have different roles, as described below.

### 4.1. Light Harvesting

Even though thin film layers of CDs and GDs have been used more as selective contacts in molecular solar cells, several groups have tried using these materials as light harvesting components. For example, Dutta and coworkers sensitized ZnO nanowires with graphene dots to prepare the structure of AZO/ZnO nanowires/GDs/TPD/Au. Graphene quantum dots participated in the charge transfer to the ZnO nanowires (nw). This is reflected in the increase of Jsc and Voc compared to the control (ZnO nw without dots) and has an efficiency of 0.2%. This low value was attributed to inefficient hole collection by TPD originated by the non-optimized thickness of the graphene layer [40]. For the deposition of GD into mesoporous layers of titania, Yan and colleagues prepared large dots functionalized with 1,3,5-trialkyl -substituted phenyl moieties (at the 2-position) at the edges of the dots to favour solubilization into common solvents and avoid aggregation. The Voc and FF of the as-prepared TiO_2_/GDs/I_3_^−^/I^−^ diodes were comparable to those obtained with the widely used Ru-based sensitizer (0.48 V, 58%, respectively). However, Jsc was much lower, which was attributed to the low affinity of the dots to the oxide surface, which resulted in poor physical adsorption and subsequent poor charge injection [36]. In addition, the dot size may have prevented effective packaging on the surface. Taking into account that graphene dots have a limited spectral absorption range in the visible, co-sensitization of the device with dyes to cover all the visible range of the spectrum emerges as an effective alternative. In this regard, the work of Fang and colleagues combined GDs with the well-known N719 dye. The dots, synthesized by acidic and hydrothermal methods from graphene oxide, were surface-passivated with PEG so carboxyl and hydroxyl groups on the surface promoted the linkage to the titania surface. Tests done with different concentrations of GDs showed that higher loadings resulted in agglomeration. The best results gave an efficiency of 6.1% due to higher Jsc and Voc than the reference, which gave 5.1% [47]. In a second example provided by Mihalache et al., N3 was combined with GDs prepared by microwave-assisted synthesis. They used this method to obtain dots with higher quantum yields and a self-passivated surface with amino functional groups to improve the affinity for the titania surface. The resulting device had better Jsc than the TiO_2_/N3 devices due to the expansion of the absorption range, which was confirmed by the increase in the IPCE throughout the range. However, the Voc was lower, although the overall efficiency of 2.15% was higher than the 1.92% of the reference. The efficiency improved as a result of the crossover between two mechanisms: first, a Foster Resonance Energy Transfer (FRET) dominant process in the blue part of the spectrum because of the significant overlap between the emission spectra of the GDs and the absorption spectrum of N3, and second, a charge transfer mediated by GDs towards the red part of the spectrum due to the cascaded energy level alignment of the LUMO levels of N3-GDs-TiO_2_ (2.98, 3.16, 4 eV, respectively), which increased the rate of electron injection [48]. Photovoltage decay analyses confirmed the hypothesis that the GDs inhibited the back electron transfer from N3 to the electrolyte. Therefore, the dots were playing a dual role in these devices as active absorbers and reductors of the recombination reactions.

Li and coworkers tested the GDs as alternatives to the popular fullerene derivative acceptors for application in organic solar cells. They reported the preparation of monodisperse graphene dots by electrochemical methods between 3 and 5 nm in size. Surface groups such as hydroxyl, carbonyl and carboxylic acid groups facilitated dispersion in common organic solvents and subsequent mixing with polymers leading to the structure ITO/PEDOT:PSS/P3HT-GQD/Al [39]. The value of the LUMO level (4.2–4.4 eV) of the GDs led to the formation of an electron transport cascade in the system P3HT-GDs-Al. Compared to P3HT-only devices, the GDs increased the exciton separation and carrier transport leading to an efficiency of 1.28%. However, the efficiency was lower than that of devices prepared with fullerenes as electron acceptors because of lower electron affinity and the non-optimized morphology, which resulted in lower FF. Similar experiments made by Gupta et al. compared the effect of graphene dots, synthesized by acidic and hydrothermal methods, and graphene sheets, both functionalized with aniline, as electron acceptors in the structure ITO/PEDOT:PSS/P3HT:ANI-GQD/LiF/Al [46]. They combined P3HT with increasing amounts of GDs in order to optimize the devices. Results were best with 1 wt. % for which efficiencies were 1.14%. Dots gave higher Jsc values than graphene sheets because their homogenous and uniform distribution within P3HT enhanced exciton separation and transport towards electrodes, which resulted, in turn, in higher FF. Another paper by Kim et al. compared the effects of GDs with different oxidation degrees on OSC [45]. The dots, prepared using Hummers’ method, were oxidized, and then hydrothermally reduced for 5 h or for 10 h before being added to the PTB7:PC71BM bulk heterojunction. During reduction, the oxygen-related functional groups were gradually removed while the size remained unaltered below 1 nm. In addition, the reduction had a negative effect on the light absorption but enhanced conductivity. After optimizing the concentration of dots in the BHJ, the researchers found that the positive effect of GDs varies with their reduction time, because Jsc increased with the oxidized dots whereas FF increased with the dots reduced for 5 h. This agreed with the observations made about the morphology and composition of the dots and shows that the functional groups, richer in oxidized GDs, play a positive role in light harvesting while sp2 carbon-richer reduced samples make a beneficial contribution to charge conductivity, decreasing the leakage current and enhancing shunt resistance and FF. The maximum efficiency, 7.6%, was thus achieved with 5 h-reduced GDs.

Finally, Tsai et al. combined GDs with n-type silicon heterojunction solar cells to expand the spectral range absorption and decrease the number of wasted photons in the UV region. To do so, they added GDs at different concentrations by solution processing on top of Ag/ITO/a-Si/ITO/Ag devices where the silicon wafer is structured as a micro pyramid [37]. The results demonstrated that the addition of GDs increased the Jsc and the FF, reaching a record efficiency of 16.55% when 0.3 wt. % concentration was used.

### 4.2. Counterelectrode

Platinum is the most popular material used as the counterelectrode in DSSC because its energy levels are suitable and it is easy to prepare. However, platinum is a rare precious metal and this increases the cost of the device. It is, therefore, prone to be substituted. In this regard, graphene sheets emerge as an excellent alternative nanomaterial because of their high carrier mobility, surface area and optical transparency. Examples of the use of plain graphene or composites of graphene with polymers, metals or carbon nanotubes can be found in the review by Wang et al. [70]. The defects and the functional groups of the sheets play a critical role in the electrocatalytic sites of the counterelectrode, making research on this topic necessary if understanding and efficiencies are to be increased. Chen and coworkers proposed a composite made of GDs embedded in polypyrrol (PPy) in the structure FTO/TiO_2_/N719/I_3_^−^/I^−^/GD-PPy as an effective method to lower the cost of the device. PPy is cheap and easy to produce although high charge transfer resistance has prevented it from being used in optoelectronic devices. Graphene dots containing −COOH and −OH groups on the edge interacted electrostatically with the N sites of the pyrrol, giving rise to highly porous structures. Cells were built with amounts of GDs ranging between 3% and 30%. Performance was best with 10%. The efficiency reached 5.27%, which is 20% more than when the pure PPy counterelectrode was used and is lower than when the electrode was Pt (efficiencies 4.46% and 6.02%, respectively). The amount of GD had to be finely tuned since increasing concentrations at low values increased the Jsc and the FF by reducing the internal resistance and enhancing charge transfer, whereas higher doping rates increased the charge recombination at the counterelectrode resulting in lower Jsc and Voc values [44].

### 4.3. Hole Collector

GDs such as HTL have been added to silicon solar cells and polymer solar cells because of their excellent charge transport properties and transparency. Since the fabrication of large area graphene sheets involves complicated deposition and transfer processes, research has also focused on solution processed GDs. Recently, Gao and coworkers reported the structure In-Ga/c-Si/GD/Au in which dots were prepared from graphene sheets with final sizes ranging between 2 and 6 nm. Epoxy, carboxyl and other oxygenous functional groups have been detected in the edges. c-Si was also passivated to improve the interaction between the two materials. Of all the options the methyl group showed the best results due to the reduction of surface carrier recombination. The diodes were prepared in air by solution processing and gave an efficiency of 6.63% which is higher than the 2.26% obtained without GDs. The dots increased Jsc and Voc because the current leakage reduced after recombination was suppressed at the anode. Although the GDs show strong absorption in the UV, the contribution to the Jsc could not be observed when the EQE was measured [42]. Moreover, the addition of GDs resulted in good stability of the c-Si/GDs cells after storage for half a year. Tsai et al. added an extra layer of PEDOT:PSS and GDs to micro-structured amorphous silicon heterojunctions leading to the configuration Al/a-Si/PEDOT:PSS-GDs/Ag. The dots, prepared by microwave methods, measured 2.9 nm, roughly 12 layers of graphene. The Jsc and FF of the diodes increased with increasing concentrations of GDs up to 0.5%, at which point the efficiency started to decline because of increased recombination reactions probably arising from the formation of GD aggregates. Therefore, a record performance of 13.22% was achieved due to the contribution of the GDs to light harvesting below 400 nm and the improvement in conductivity and the subsequent carrier collection efficiency [41].

Searching for enhanced stability and lifetime, Li and colleagues used GDs in polymer solar cells to substitute the hygroscopic PEDOT:PSS in the configuration ITO/GDs/P3HT:PCBM/LiF/Al. The dots were created by acid treatment of carbon fibers. Optimization of the HTL thickness between 1.5 and 2 nm resulted in devices that had efficiency values similar to those of the cells prepared with PEDOT:PSS, 3.5%, due to the homogeneous morphology and good conductivity of the GDs. Moreover, measurements of efficiency in air showed that decay was slower when GDs were used. The same experiments performed on small molecule solar cells based on DR3TBDT:PC71BM gave efficiencies similar to PEDOT:PSS containing devices (6.9% efficiency), thus demonstrating the capability of GDs to act as a hole collector [50].

### 4.4. Electron Collector

**Figure 7:**
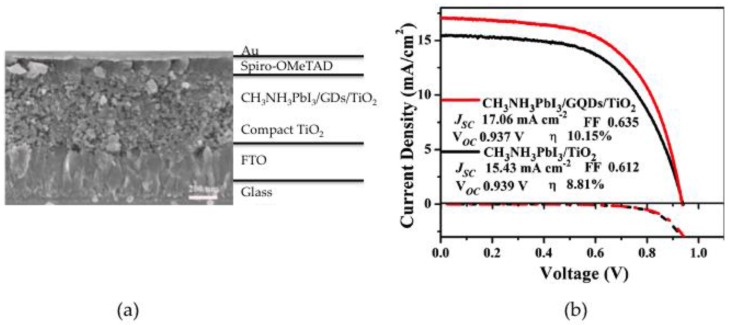
(**a**) Cross-sectional view of the SEM image; (**b**) JV curve of the devices comparing the effect of the insertion of the GDs. Reproduced with permission of [38].

Perovskite-based solar cells have recently attracted the research community because of their broad spectral absorption and conducting properties. These molecules have been applied in planar and mesoscopic heterojunctions and have shown efficiencies over 22% [3,71]. Meanwhile, GDs have shown ultrafast hot-electron extraction faster than 15 fs through the GDs-TiO_2_ interface [72], although their application in DSSC has given low efficiencies. However, to further improve performance, Zhu et al. inserted an ultrathin layer of GDs between the perovskite and the titania layer in the configuration FTO/TiO_2_ dense/TiO_2_ mesoporous/CH_3_NH_3_PbI_3_/GDs/spiro-OMeTAD/ Au (Figure 7). The dots, prepared by electrochemical methods, measured between 5 and 10 nm and were homogeneously distributed onto the titania layer. Optimization of the thin layer thickness led to efficiencies of 10.15%, which is higher than the 8.81% reported for the reference cell without GDs. Whereas the FF and Voc showed values similar to the reference, the Jsc increased due to faster charge extraction. Involvement of the GDs in light harvesting was discarded since the strong absorption of the perovskite dominates absorption and no contribution from the GDs could be detected [38].

## 5. Carbon Dots in Photovoltaics

The light harvesting abilities and conducting properties of carbon dots have prompted researchers to use them in a variety of roles in solar cells.

### 5.1. Light Harvesting

The spectral absorption features of the carbon dots in the ultraviolet region have led to their application as single absorbers in several photovoltaic cells. Briscoe and co-workers studied the construction of low-cost sustainable structured cells making use of carbon dots (CDs) obtained from biomass. They prepared the dots by hydrothermal carbonization of chitin, chitosan or glucose which led to samples with features that reflected the parent reactant. Thus, chitin and chitosan led to *N*-doped CDs (10% and 8% doping, respectively). The surface was functionalized by amides if chitin was used, amines if chitosan was used and hydroxyl if glucose was used. The differences remained during deposition onto ZnO nanorods because the best coverages were obtained with chitosan and glucose. Finally, CuSCN was added as HTL giving rise to the cell configuration FTO/ZnO nanorod/CDs/CuSCN/Au. Efficiencies were best (0.061%) with chitosan-derived CDs. It was observed that the nature of the precursor and surface functionalization heavily influences the performance of the diodes. For further optimization, the authors combined two types of CD to merge their best properties and increase optical absorption. However, the combination needed to be done with great care to prevent the series resistance from increasing and the Jsc from decreasing. Therefore, results were best with a combination of chitosan and chitin-derived carbon dots, for which efficiency was 0.077 [29]. 

Mirtchev et al. explored CD- DSSC with mesoporous titania. The dots were prepared by dehydratation of γ-butyrolactone and contained sulfonate, carboxylate and hydroxyl groups on the surface, thus mimicking the anchoring groups of common Ru-based sensitizers. The device was built by immersing titania in CD solution for 48 h and was completed with I_3_^−^/I^−^ as HTL to give the structure FTO/TiO_2_/CDs/I_3_^−^/I^−^/P [22]. In comparison with typical Ru-sensitizers, Jsc is the factor that limits better efficiencies because of the emissive trap sites on the surface of the dot that could act as recombination centers and because of the lower capacity of the dot to inject charges into TiO_2_. The authors suggested maximizing the titania surface coverage by using smaller dots or bifunctional linker molecules to enhance the efficiency [22]. Sun et al. used a similar device configuration with titania nanotubes. The dots were prepared by electrochemical-etching methods and added to the nanotubes by impregnation for several hours. Assembly between the small dots and the titania was possible through the oxygen functional groups present on the surface of the carbon material. The device, which has a low efficiency of 0.0041%, served as proof-of-concept of the light harvesting properties of the CDs. The authors expected that optimizing the electrolyte and the electrodes would give better results [28].

Zhang et al. developed hierarchical microspheres of rutile built by uniform nanorods to prepare solar cells made of metal-free sensitizers. They synthesized nitrogen-doped carbon dots (NCDs) by one-pot solvothermal methods and anchor them to the rutile structures by means of the surface groups. The configuration of the cell was TiO_2_/NCDs/I_3_^−^/I^−^/Pt and the Jsc values were higher than those of similar devices prepared without NCDs. The final efficiency was 0.13% [25].

**Figure 8:**
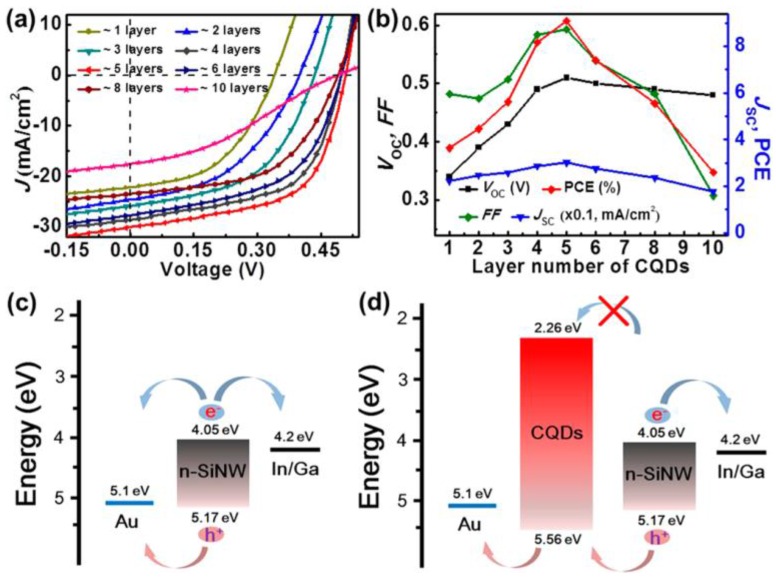
Variation of the JV curve (**a**) and cell parameters (**b**) with increasing layers of CDs; Energy level alignments of the cells without (**c**) and with (**d**) CDs. Reproduced with permission of [30].

CDs have also been applied in nanostructured silicon solar cells. Xie et al. intended to broaden the absorption range of the silicon nanowires (Si nw) by creating core/shell heterojunctions with carbon dots. The nanoparticles were synthesized by electrochemical etching methods and added to the silicon wires to form a homogeneous and continuous shell of 23 nm corresponding to 5 layers of dots. The overall structure of the device was In-Ga/Si nw/CD/Au and reached an efficiency of 9.1% which is much higher than the references prepared with planar silicon and five layers of CDs (4.05%) or silicon nanowires without CDs (1.58%) [30]. The reasons for the enhanced performance of the device were the increase in optical absorption in the UV region and the fact that recombination was lower because of the electron blocking layer action of the CDs (Figure 8).

An innovative approach has recently been reported by Huang et al. who prepared composites of CDs and polysiloxane to coat the substrate of the solar cells, which had the configuration CD-polysiloxane/ITO/ZnO/P3HT:PCBM/MoO_3_/Ag [31]. The dots were prepared by a one-step reaction with ascorbic acid as the carbon source and KH791 as the stabilizing and passivating agent and source of the siloxane polymer. The composite contributed to light harvesting in the UV part of the spectrum and increased the efficiency by about 12% compared to the polymer:fullerene solar cell (3.18% and 2.85%, respectively). Similar observations are reported by Liu et al. who added increasing amounts of CDs to the active layer in the cell configuration ITO/TiO_2_/PCDTBT:PCBM:CDs/MoO_3_/Ag. The increase in absorption in the UV region, together with the improvement in charge transport resulted in enhanced FF and Jsc when 0.062 wt. % ratio was used leading to efficiencies of 7.05% [26].

### 5.2. Counterelectrode

Dao et al. studied different options for the counterelectrode (CE) component of quantum dot solar cells looking for lower resistance and higher reduction rates of the redox electrolyte. They compared sputtered gold, CDs and CD-containing gold particles in the ZnO nanowire/CdS/CdSe/polysulfide electrolyte/CE configuration. The Cd-Au structures were formed by a dense array of gold rods covered by small 1.2 nm CDs in a 200 nm wide raspberry-like superstructure. When applied as the CE, they showed enhanced redox activity toward the polysulfide electrolyte that increased the efficiency to 5.4% whereas CDs and the sputtered gold gave efficiencies of 0.18 and 3.6%, respectively. These results are explained by the larger surface area of the Au-CD structures and the reduced internal charge transfer resistance of the material that contributed to the increment of Jsc and the FF [27].

### 5.3. Hole Collection

CDs have also been tested in the charge transport layers of perovskite solar cells as alternatives to the expensive hole transporter spiro-OMeTAD in the configuration FTO/TiO_2_ dense/TiO_2_ mesoporous/CH_3_NH_3_PbI_3-x_Cl_x_/CDs/Au [35]. The dots were prepared by polymerization-carbonization of citric acid using p-phenylenediamine as passivating agent and deposited by solution processing onto the perovskite layer. The resulting devices performed better than the control without HTL, although Jsc, Voc and FF values were lower than those of the spiro-OMeTAD device. The poorer performance (3% vs. 8% efficiencies for the CDs and the spiro-OMeTAD-containing devices, respectively) was attributed to non-optimized device fabrication.

### 5.4. Electron Collection

The potential contribution of CDs to the charge transport in the solar cells led to the nanocrystals being used as electron acceptors. Kwon and coworkers tested oleylamine-capped CDs in combination with the electron donor P3HT to form the structure ITO/PEDOT:PSS/P3HT:CDs/Al [32]. Compared to the 1.99% efficiency of the P3HT/PCBM reference, the 0.23% obtained points to the insulating character of oleylamine as the origin of the lower Jsc values. Zhang et al. in addition, worked on organic solar cells and tested the ability of the CDs as electron acceptors. They prepared the configuration ITO/PEDOT:PSS/DR3TBDT:PC71BM/ETL/Al (ETL: electron transport layer) and observed that the efficiency of the devices increased to 7.67% when CDs replaced the widely used LiF in the ETL. In addition, extended lifetimes due to the air stability of the dots were also reported. When the small molecular light harvesters were replaced by P3HT:PCBM, the efficiency was also higher when CDs were used instead of LiF (3.42% vs. 3.38%, respectively) [23]. The improvement was attributed in both cases to the balance of the charge transport by decreasing the series resistance and increasing the shunt resistance resulting in the increase of charge collection.

**Figure 9:**
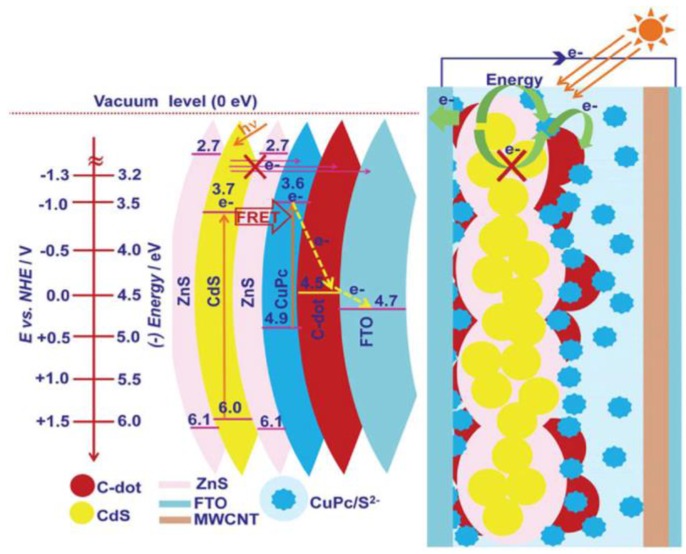
Energy band diagram showing possible paths for energy and charge transport and structure of the FTO/ZnS-CdS-ZnS/CDs/CuPc/S^2−^/MWCNT devices. Reproduced with permission of [33].

Another strategy for enhancing the charge transport is to combine CDs with electron acceptor molecules. Narayanan et al. described a device made of quantum dots ZnS/CdS/ZnS, which act as an exciton generator, and the small molecule CuPc as an electron acceptor (Figure 9). The quantum dots absorbed light in the blue-green region of the spectrum and transferred the energy via Förster resonance to the red absorber phthalocyanine. The addition of the CDs to the heterojunction accelerated the charge transfer towards the electrode and decreased the electron recombination rate, which was reflected in the increase in IPCE. Thus, the resulting Jsc was 5.76 times higher than the reference prepared without CDs. Voc was also enhanced, and the efficiency increased to 0.35%. The carbon nanocrystals measured 16 nm and were closely connected to CDs and CuPc, as observed by HRTEM [33]. 

Similar results were observed by Ma et al. when they added CDs to titania functionalized with the rhodamine B sensitizer in the system FTO/TiO_2_/RhB/CQD/I_3_^−^/I^−^/Pt [34]. The combination of the dots with rhodamine increased light harvesting in the UV region and suppressed electron recombination leading to 0.147% efficiency. Therefore, electrochemically generated CDs were responsible for the 7-fold increase in the Jsc.

## 6. Outlook and Perspectives

Carbon-based materials are an exciting challenge in the area of materials chemistry and nanotechnology. Needless to say, they are abundant and they are also inert, non-toxic and, when scaled-up, cost effective. However, at present their applications are limited due to the numerous physical and chemical phenomena that are still unexplored. This review aimed to give a general overview of the enormous potential graphene and carbon dots have in photovoltaic applications. There are, of course, more applications, but the ones discussed here will help researchers interested in exploring the boundaries of graphene and carbon nanoform research. 

For instance, the absorption ability of the carbon nanostructures in the UV region complements light harvesting in those cells where absorption is confined to the visible region. The increased number of captured photons leads to the boost of the IPCE and the Jsc. On the other hand, their redox characteristics accelerate charge transfer from the absorber to the electrode. Therefore, electron recombination diminishes whereas Voc increases. These beneficial effects are influenced by the synthetic approach, which determines the size of the particles and the functional groups found on the surface and edges of the crystals. These groups have a major influence on the optical properties and the interactions with the materials of which the devices are made. In this regard, some authors have investigated on the addition of specific functionalities to enhance the interaction between the dots and other components of the device. However, the synthetic variability hinders reproducibility and affects the efficiency. The examples reported in this review highlight the need for further optimization of the structure and linkage; so consequently, the size and surface molecules need to be fine tuned if efficient devices are to be prepared. Nonetheless, these materials are expected to play an important role in energy-harvesting devices that help to decrease CO_2_ emissions and lower the cost of renewable energy.

## Figures and Tables

**Table 1:** Summary of the synthetic techniques of CDs included in this article, the resulting size and functional groups and the performance of the photovoltaic cells in which they are used.

Synthesis ^1^	Carbon Source	Average Size (nm)	Surface Groups	Solar Cell ^2^	Jsc (mA/cm^2^)	Voc (V)	FF (%)	η (%)	Effect	R ^3^
H	γ-butyrolactone	9 ± 6	Sulfonate, carboxyl, hydroxyl, alkyl	DSSC	0.53	0.38	64	0.13	Emissive traps on the dot surface and enhancement of recombination	[22]
H	Citric acid	1–2	carboxyl	SMOPV	13.32	0.904	63.7	7.67	Increment in exciton separation and charge collection	[23]
				PSC	9.98	0.609	54.8	3.42		
H	CCl_4_	1.5–3.3	Amino, carboxylic	DSSC	0.33	0.370	43	0.13	Contribution to light absorption	[24,25]
H	Polystyrene-co-maleic anhydride	---	---	PSC	13.61	0.870	59.5	7.05	Improvement of absorption in the UV and charge transport	[26]
M	Citric acid	200 ^4^	Carboxylic, primary amines	QDSC	16.6 ^4^	0.708 ^4^	46 ^4^	5.4 ^4^	Improved charge extraction	[27]
		1.2 ^5^			2.0 ^5^	0.550 ^5^	16 ^5^	0.18 ^5^		
E	Graphite rods	<4	Hydroxyl, carboxyl, aromatic groups, epoxide/ether	DSSC	0.02	0.580	35	0.0041	Non-optimized electrolyte and electrode	[28]
H	Biomass (chitin, chitosan, glucose)	14.1 ± 2.4 chitin 8.1 ± 0.3 chitosan 2.57 ± 0.04 glucose	Amine, amide, hydroxyl	DSSC	0.674 ^6^	0.265 ^6^	43 ^6^	0.077 ^6^	Influence of surface groups	[29]
E	Graphite rod	4.5	---	Si	30.09	0.510	59.3	9.1	Improvement of absorption in the UV and decrease of recombination	[30]
H	Ascorbic acid	3–4	Carboxylic, hydroxyl	DSSC	8.40	0.610	62	3.18	Improvement of light absorption	[31]
S	Citric acid	1.5	Aldehyde, carboxylic	PSC	0.288	1.588	48.5	0.23	Insulating character of oleylamine ligand	[32]
H	Glucose	16	---	QDSC	1.88	0.605	31	0.35	Increment of charge transfer and decrease of recombination	[33]
E	Graphite rods	<10	---	DSSC	0.64	0.500	--	0.147	Improvement of absorption in the UV and decrease of recombination	[34]
H	Citric acid	2–3	---	PerSC	7.83	0.515	74	3.00	Non-optimized device	[35]

^1^ H: hydrothermal, M: microwave, E: electrochemical, S: soft template synthesis; ^2^ DSSC: dye-sensitized solar cell, SMOPV: small molecule organic solar cell, PSC: polymer-based organic solar cells, Si: silicon-based solar cell, QDSC: quantum dot sensitized solar cell, PerSC: perovskite-based solar cell; ^3^ R: reference; ^4^: CDs-Au particles; ^5^: CDs; ^6^ best results obtained from the combination of chitosan- and chitin-derived CDs.

**Table 2:** Summary of the synthetic techniques of GDs included in this article, the resulting size and functional groups and the performance of the photovoltaic cells in which they are used.

Synthesis ^1^	Carbon Source	Size (nm)	Surface Groups	Solar Cell ^2^	Jsc (mA/cm^2^)	Voc (V)	FF (%)	η (%)	Effect	R^3^
H	Bromobenzoic acid	13.5	1,3,5 trialkyl phenyl	DSSC	0.2	0.48	58	0.055	Poor charge injection due to low affinity of GDs to titania	[36]
M	Glucose	3.4	---	Si	37.47	0.61	72.51	16.55	Improvement of absorption in the UV	[37]
E	Graphite rod	5–10	Hydroxyl, epoxy, carboxylic, carbonyl	PerSC	17.06	0.937	63.5	10.15	Improvement of charge extraction	[38]
E	Graphene film	3–5	Hydroxyl, carbonyl	PSC	6.33	0.67	30	1.28	Increment of exciton separation and charge transport. Non-optimized morphology	[39]
A	Graphite	8.5	---	DSSC	0.45	0.8	50	0.2	Inefficient hole collection due to non-optimized thickness of GD layer	[40]
M	Glucose	2.9	---	Si	36.26	0.57	63.87	13.22	Improvement of absorption in the UV and conductivity	[41]
A+H	Graphene oxide	2–6	Epoxy, carboxyl	Si	23.38	0.51	55	6.63	Reduction in current leakage	[42,43]
A	Carbon black	10	Hydroxyl, carboxyl	DSSC	14.36	0.723	50.8	5.27	Reduction in internal resistance and increment of charge transfer	[44]
A+H	Graphene oxide	<1	Epoxy, carbonyl, hydroxyl	PSC	15.2	0.74	67.6	7.6	Increment in conductivity	[45]
A+H	Graphene sheets	9	Carboxyl ^4^	PSC	3.51	0.61	53	1.14	Increase in exciton separation and charge transport	[46]
A+H	Graphene oxide	50	PEG	DSSC	14.07	0.66	59	6.1	Increase in light absorption	[47]
M	Glucosamine hydrochloride	4.3	amine	DSSC	5.58	0.583	66	2.15	Increase in light absorption and decrease of recombination	[48]
A	Carbon fibers	20–30	---	PSC	10.2	0.52	66.3	3.5	Increase in conductivity	[49,50]
				SMOPV	11.36	0.92	65.2	6.82		

^1^ H: hydrothermal, M: microwave, E: electrochemical, A: acidic oxidation; ^2^ DSSC: dye sensitized solar cell, SMOPV: small molecule organic solar cell, PSC: polymer-based organic solar cells, Si: silicon-based solar cell, QDSC: quantum dot sensitized solar cell, PerSC: perovskite-based solar cell; ^3^ R: reference; ^4^ surface groups attached after chemical treatment.

## Abbreviations

The following abbreviations are used in this manuscript:

AFM	Atomic force microscope
AZO	Aluminum-doped zinc oxide
CDs	Carbon quantum dots
DSSC	Dye-sensitised solar cells
DR3TBDT	3-ethyl rhodanine benzo[1,2-b:4,5-b′]dithiophene
ETL	Electron transport layer
GDs	Graphene dots
HTL	Hole transport layer
HRTEM	High resolution transmission electron microscopy
KH791	(N-(2-aminoethyl)-3-aminopropyl)tris-(2-ethoxy) silane
N719	Di-tetrabutylammonium cis-bis(isothiocyanato) bis (2,2′-bipyridyl -4,4′- dicarboxylato) ruthenium(II)
N3	Cis-Bis(isothiocyanato) bis(2,2′-bipyridyl-4,4′-dicarboxylato ruthenium(II)
MWCNT	Multiwall carbon nanotubes
NCDs	Nitrogen-doped carbon dots
OSC	Organic solar cells
P3HT	Poly(3-hexyl thiophene)
PEDOT:PSS	Poly(3,4-ethylenedioxythiophene) polystyrene sulfonate
PCBM	[6,6]-phenyl-C61-butyric acid methyl ester
PffBT4T-2OD	Poly [(5,6-difluoro-2,1,3-benzothiadiazol-4,7-diyl)-alt-(3,3‴–di (2-octyldodecyl)-2, 2′; 5′, 2″; 5″, 2‴-quaterthiophen-5,5‴-diyl)
Ppy	Polypyrrol
PSC	Polymer solar cell
SMOPV	Small molecule organic photovoltaics
TC71BM	[6,6]-2-Thienyl-C71-butyric acid methyl ester
TPD	NN′-diphenyl-N-N′-bis(3-methylphenyl)-1,1′-biphenyl)-4,4′-diamine
Spiro-OMeTAD	N2,N2,N2′,N2′,N7,N7,N7′,N7′-octakis(4-methoxyphenyl)-9,9′-spiro bi[9H-fluorene]-2,2′,7,7′-tetramine

## References

[1] Armaroli N., Balzani V. (2016). Solar Electricity and Solar Fuels: Status and Perspectives in the Context of the Energy Transition. Chem. A Eur. J..

[2] Green M.A., Emery K., Hishikawa Y., Warta W., Dunlop E.D. (2016). Solar cell efficiency tables (version 47). Prog. Photovolt. Res. Appl..

[3] National Center for Photovoltaics. http://www.nrel.gov/ncpv/.

[4] Albero J., Clifford J.N., Palomares E. (2014). Quantum dot based molecular solar cells. Coord. Chem. Rev..

[5] Medintz I.L., Uyeda H.T., Goldman E.R., Mattoussi H. (2005). Quantum dot bioconjugates for imaging, labelling and sensing. Nat. Mater..

[6] Nurmikko A. (2015). What future for quantum dot-based light emitters?. Nat. Nanotechnol..

[7] Xu X., Ray R., Gu Y., Ploehn H.J., Gearheart L., Raker K., Scrivens W.A. (2004). Electrophoretic analysis and purification of fluorescent single-walled carbon nanotube fragments. J. Am. Chem. Soc..

[8] Sun Y.P., Zhou B., Lin Y., Wang W., Fernando K.A.S., Pathak P., Meziani M.J., Harruff B.A., Wang X., Wang H. (2006). Quantum-sized carbon dots for bright and colorful photoluminescence. J. Am. Chem. Soc..

[9] Novoselov K.S., Geim A.K., Morozov S.V., Jiang D., Zhang Y., Dubonos S.V., Grigorieva I.V., Firsov A.A. (2011). Electric Field Effect in Atomically Thin Carbon Films. Science.

[10] Ding C., Zhu A., Tian Y. (2014). Functional surface engineering of C-dots for fluorescent biosensing and in vivo bioimaging. Acc. Chem. Res..

[11] Li H., Kang Z., Liu Y., Lee S.-T. (2012). Carbon nanodots: Synthesis, properties and applications. J. Mater. Chem..

[12] Zheng X.T., Ananthanarayanan A., Luo K.Q., Chen P. (2015). Glowing graphene quantum dots and carbon dots: Properties, syntheses, and biological applications. Small.

[13] Baker S.N., Baker G.A. (2010). Luminescent carbon nanodots: Emergent nanolights. Angew. Chem. Int. Ed..

[14] Shen J., Zhu Y., Yang X., Li C. (2012). Graphene quantum dots: Emergent nanolights for bioimaging, sensors, catalysis and photovoltaic devices. Chem. Commun..

[15] Ding H., Yu S.-B., Wei J.-S., Xiong H.-M. (2015). Full-Color Light-Emitting Carbon Dots with a Surface-State-Controlled Luminescence Mechanism. ACS Nano.

[16] Wang Y., Hu A. (2014). Carbon quantum dots: Synthesis, properties and applications. J. Mater. Chem. C.

[17] Li X.M., Rui M.C., Song J.Z., Shen Z.H., Zeng H.B., Li X.M., Rui M.C., Song J.Z., Shen Z.H., Zeng H.B. (2015). Carbon and Graphene Quantum Dots for Optoelectronic and Energy Devices: A Review. Adv. Funct. Mater..

[18] Zhang Z., Zhang J., Chen N., Qu L. (2012). Graphene quantum dots: An emerging material for energy-related applications and beyond. Energy Environ. Sci..

[19] Miao P., Han K., Tang Y., Wang B., Lin T., Cheng W. (2015). Recent advances in carbon nanodots: Synthesis, properties and biomedical applications. Nanoscale.

[20] Fan Z., Li S., Yuan F., Fan L. (2015). Fluorescent graphene quantum dots for biosensing and bioimaging. RSC Adv..

[21] Van Pham C., Madsuha A.F., Nguyen T.V., Krueger M. (2016). Graphene-quantum dot hybrid materials on the road to optoelectronic applications. Synth. Met..

[22] Mirtchev P., Henderson E.J., Soheilnia N., Yip C.M., Ozin G.A. (2012). Solution phase synthesis of carbon quantum dots as sensitizers for nanocrystalline TiO_2_ solar cells. J. Mater. Chem..

[23] Zhang H., Zhang Q., Li M., Kan B., Ni W., Wang Y., Yang X., Du C., Bc X.W., Chen Y. (2015). Investigation of the enhanced performance and lifetime of organic solar cells using solution-processed carbon dots as the electron transport layers. J. Mater. Chem. C.

[24] Zhang Y.-Q., Ma D.-K., Zhuang Y., Zhang X., Chen W., Hong L.-L., Yan Q.-X., Yu K., Huang S.-M. (2012). One-pot synthesis of N-doped carbon dots with tunable luminescence properties. J. Mater. Chem..

[25] Zhang Y.Q., Ma D.K., Zhang Y.G., Chen W., Huang S.M. (2013). N-doped carbon quantum dots for TiO_2_-based photocatalysts and dye-sensitized solar cells. Nano Energy.

[26] Liu C., Chang K., Guo W., Li H., Shen L., Chen W., Yan D. (2014). Improving charge transport property and energy transfer with carbon quantum dots in inverted polymer solar cells. Appl. Phys. Lett..

[27] Dao V.D., Kim P., Baek S., Larina L.L., Yong K., Ryoo R., Ko S.H., Choi H.S. (2016). Facile synthesis of carbon dot-Au nanoraspberries and their application as high-performance counter electrodes in quantum dot-sensitized solar cells. Carbon.

[28] Sun M., Ma X., Chen X., Sun Y., Cui X., Lin Y. (2014). A nanocomposite of carbon quantum dots and TiO_2_ nanotube arrays: Enhancing photoelectrochemical and photocatalytic properties. RSC Adv..

[29] Briscoe J., Marinovic A., Sevilla M., Dunn S., Titirici M. (2015). Biomass-Derived Carbon Quantum Dot Sensitizers for Solid-State Nanostructured Solar Cells. Angew. Chem. Int. Ed..

[30] Xie C., Nie B., Zeng L., Liang F.X., Wang M.Z., Luo L., Feng M., Yu Y., Wu C.Y., Wu Y. (2014). Core-shell heterojunction of silicon nanowire arrays and carbon quantum dots for photovoltaic devices and self-driven photodetectors. ACS Nano.

[31] Huang J.J., Zhong Z.F., Rong M.Z., Zhou X., Chen X.D., Zhang M.Q. (2014). An easy approach of preparing strongly luminescent carbon dots and their polymer based composites for enhancing solar cell efficiency. Carbon.

[32] Kwon W., Lee G., Do S., Joo T., Rhee S.W. (2014). Size-controlled soft-template synthesis of carbon nanodots toward versatile photoactive materials. Small.

[33] Narayanan R., Deepa M., Srivastava A.K. (2013). Forster resonance energy transfer and carbon dots enhance light harvesting in a solid-state quantum dot solar cell. J. Mater. Chem. A.

[34] Ma Z., Zhang Y.L., Wang L., Ming H., Li H., Zhang X., Wang F., Liu Y., Kang Z., Lee S.T. (2013). Bioinspired photoelectric conversion system based on carbon-quantum-dot- doped dye-semiconductor complex. ACS Appl. Mater. Interfaces.

[35] Paulo S., Stoica G., Cambarau W., Martinez-Ferrero E., Palomares E. (2016). Carbon quantum dots as new hole transport material for perovskite solar cells. Synth. Met..

[36] Yan X., Cui X., Li B., Li L.S. (2010). Large, solution-processable graphene quantum dots as light absorbers for photovoltaics. Nano Lett..

[37] Tsai M.-L., Tu W.-C., Tang L., Wei T.-C., Wei W.-R., Lau S.P., Chen L.-J., He J.-H. (2016). Efficiency Enhancement of Silicon Heterojunction Solar Cells via Photon Management Using Graphene Quantum Dot as Downconverters. Nano Lett..

[38] Zhu Z., Ma J., Wang Z., Mu C., Fan Z., Du L., Bai Y., Fan L., Yan H., Phillips D.L. (2014). Efficiency enhancement of perovskite solar cells through fast electron extraction: The role of graphene quantum dots. J. Am. Chem. Soc..

[39] Li Y., Hu Y., Zhao Y., Shi G., Deng L., Hou Y., Qu L. (2011). An electrochemical avenue to green-luminescent graphene quantum dots as potential electron-acceptors for photovoltaics. Adv. Mater..

[40] Dutta M., Sarkar S., Ghosh T., Basak D. (2012). ZnO/graphene quantum dot solid-state solar cell. J. Phys. Chem. C.

[41] Tsai M.-L., Wei W.-R., Tang L., Chang H.-C., Tai S.-H., Yang P.-K., Lau S.P., Chen L.-J., He J.-H. (2016). 13% Efficiency Si Hybrid Solar Cells via Concurrent Improvement in Optical and Electrical Properties by Employing Graphene Quantum Dots. ACS Nano.

[42] Gao P., Ding K., Wang Y., Ruan K., Diao S., Zhang Q., Sun B., Jie J. (2014). Crystalline Si/graphene quantum dots heterojunction solar cells. J. Phys. Chem. C.

[43] Pan D., Zhang J., Li Z., Wu M. (2010). Hydrothermal route for cutting graphene sheets into blue-luminescent graphene quantum dots. Adv. Mater..

[44] Chen L., Guo C.X., Zhang Q., Lei Y., Xie J., Ee S., Guai G., Song Q., Li C.M. (2013). Graphene quantum-dot-doped polypyrrole counter electrode for high-performance dye-sensitized solar cells. ACS Appl. Mater. Interfaces.

[45] Kim J.K., Park M.J., Kim S.J., Wang D.H., Cho S.P., Bae S., Park J.H., Hong B.H. (2013). Balancing light absorptivity and carrier conductivity of graphene quantum dots for high-efficiency bulk heterojunction solar cells. ACS Nano.

[46] Gupta V., Chaudhary N., Srivastava R., Sharma G.D., Bhardwaj R., Chand S. (2011). Luminscent graphene quantum dots for organic photovoltaic devices. J. Am. Chem. Soc..

[47] Fang X., Li M., Guo K., Li J., Pan M., Bai L., Luoshan M., Zhao X. (2014). Graphene quantum dots optimization of dye-sensitized solar cells. Electrochim. Acta.

[48] Mihalache I., Radoi A., Mihaila M., Munteanu C., Marin A., Danila M., Kusko M., Kusko C. (2015). Charge and energy transfer interplay in hybrid sensitized solar cells mediated by graphene quantum dots. Electrochim. Acta.

[49] Peng J., Gao W., Gupta B.K., Liu Z., Romero-Aburto R., Ge L., Song L., Alemany L.B., Zhan X., Gao G. (2012). Graphene quantum dots derived from carbon fibers. Nano Lett..

[50] Li M., Ni W., Kan B., Wan X., Zhang L., Zhang Q., Long G., Zuo Y., Chen Y. (2013). Graphene quantum dots as the hole transport layer material for high-performance organic solar cells. Phys. Chem. Chem. Phys..

[51] Li L., Wu G., Yang G., Peng J., Zhao J., Zhu J.-J. (2013). Focusing on luminescent graphene quantum dots: Current status and future perspectives. Nanoscale.

[52] Low C.T.J., Walsh F.C., Chakrabarti M.H., Hashim M.A., Hussain M.A. (2013). Electrochemical approaches to the production of graphene flakes and their potential applications. Carbon.

[53] Zhang M., Bai L., Shang W., Xie W., Ma H., Fu Y., Fang D., Sun H., Fan L., Han M. (2012). Facile synthesis of water-soluble, highly fluorescent graphene quantum dots as a robust biological label for stem cells. J. Mater. Chem..

[54] Sk M.A., Ananthanarayanan A., Huang L., Lim K.H., Chen P. (2014). Revealing the tunable photoluminescence properties of graphene quantum dots. J. Mater. Chem. C.

[55] Song Y., Zhu S., Yang B. (2014). Bioimaging based on fluorescent carbon dots. RSC Adv..

[56] Gan Z., Xiong S., Wu X., Xu T., Zhu X., Gan X., Guo J., Shen J., Sun L., Chu P.K. (2013). Mechanism of photoluminescence from chemically derived graphene oxide: Role of chemical reduction. Adv. Opt. Mater..

[57] Hong G., Diao S., Antaris A.L., Dai H. (2015). Carbon Nanomaterials for Biological Imaging and Nanomedicinal Therapy. Chem. Rev..

[58] Gan Z., Xu H., Hao Y. (2016). Mechanism for excitation-dependent photoluminescence from graphene quantum dots and other graphene oxide derivates: Consensus, debates and challenges. Nanoscale.

[59] Ritter K.A., Lyding J.W. (2009). The influence of edge structure on the electronic properties of graphene quantum dots and nanoribbons. Nat. Mater..

[60] Xie C., Zhang X., Wu Y., Zhang X., Zhang X., Wang Y., Zhang W., Gao P., Han Y., Jie J. (2013). Surface passivation and band engineering: A way toward high efficiency graphene-planar Si solar cells. J. Mater. Chem. A.

[61] Clifford J.N., Martínez-Ferrero E., Viterisi A., Palomares E. (2011). Sensitizer molecular structure-device efficiency relationship in dye sensitized solar cells. Chem. Soc. Rev..

[62] O’Regan B., Gratzel M. (1991). A low-cost, high-efficiency solar cell based on dye-sensitized colloidal TiO_2_ films. Nature.

[63] Mathew S., Yella A., Gao P., Humphry-Baker R., Curchod B.F.E., Ashari-Astani N., Tavernelli I., Rothlisberger U., Khaja N., Grätzel M. (2014). Dye-sensitized solar cells with 13% efficiency achieved through the molecular engineering of porphyrin sensitizers. Nat. Chem..

[64] Weickert J., Dunbar R.B., Hesse H.C., Wiedemann W., Schmidt-Mende L. (2011). Nanostructured organic and hybrid solar cells. Adv. Mater..

[65] Brabec C.J., Heeney M., McCulloch I., Nelson J. (2011). Influence of blend microstructure on bulk heterojunction organic photovoltaic performance. Chem. Soc. Rev..

[66] Lu L., Zheng T., Wu Q., Schneider A.M., Zhao D., Yu L. (2015). Recent Advances in Bulk Heterojunction Polymer Solar Cells. Chem. Rev..

[67] Liu Y., Zhao J., Li Z., Mu C., Ma W., Hu H., Jiang K., Lin H., Ade H., Yan H. (2014). Aggregation and morphology control enables multiple cases of high-efficiency polymer solar cells. Nat. Commun..

[68] Zhao J., Li Y., Yang G., Jiang K., Lin H., Ade H., Ma W., Yan H. (2016). Efficient organic solar cells processed from hydrocarbon solvents. Nat. Energy.

[69] Zhao W., Qian D., Zhang S., Li S., Inganäs O., Gao F., Hou J. (2016). Fullerene-Free Polymer Solar Cells with over 11% Efficiency and Excellent Thermal Stability. Adv. Mater..

[70] Wang H., Hu Y.H. (2012). Graphene as a counter electrode material for dye-sensitized solar cells. Energy Environ. Sci..

[71] Yang W.S., Noh J.H., Jeon N.J., Kim Y.C., Ryu S., Seo J., Seok S. (2015). High-performance photovoltaic perovskite layers fabricated through intramolecular exchange. Science.

[72] Williams K.J., Nelson C.A., Yan X., Li L.-S., Zhu X. (2013). Hot Electron Injection from Graphene Quantum Dots to TiO_2_. ACS Nano.

